# Olfactory Assessment of Competitors to the Nest Site: An Experiment on a Passerine Species

**DOI:** 10.1371/journal.pone.0167905

**Published:** 2016-12-09

**Authors:** Matteo Griggio, Gerardo Fracasso, Katharina Mahr, Herbert Hoi

**Affiliations:** 1 Dipartimento di Biologia, Università di Padova, Via U. Bassi Padova, Italy; 2 Konrad Lorenz Institute of Ethology, Department of Integrative Biology and Evolution, University of Veterinary Medicine of Vienna, Vienna, Austria; Universidad de Granada, SPAIN

## Abstract

Since most avian species have been considered anosmic or microsmatic, olfaction and associated behavioural patterns have hardly been investigated. Most importantly, empirical data on avian olfaction is not equally distributed among species. Initial investigations focused on species with relatively big olfactory bulbs because they were thought to have better olfactory capabilities. Hence, in this study we tested the ability of house sparrows (*Passer domesticus*) to use chemical cues as parameters to estimate nest features. House sparrows are a commonly used model species, but their olfactory capabilities have not been studied so far. We offered two different odours to males and females, namely the scent of mouse urine (*Mus musculus domesticus*), representing a possible competitor and a threat to eggs and hatchlings, and the odour of hay, representing a familiar and innocuous odour. The experiment was performed at the sunset to simulate a first inspection to new possible roosting or nesting sites. Interestingly, males but not females preferred to spend significantly more time in front of the hay odour, than in front of the scent of mouse urine. Our results strengthen the hypothesis that oscines can not only perceive odours but also use olfaction to assess the environment and estimate nest site quality.

## Introduction

Only within the last decades have a substantial number of studies started to investigate the use of chemical cues in regard to a variety of functional contexts in avian species such as homing behaviour [1–3], foraging [4–7], mate choice [8,9], kinship recognition [10,11], and predator detection [12–15]. In particular, olfactory predator detection has only recently been shown to be a capability present even in those passerine species that were considered to have relatively small olfactory systems [12–16].

Among avian taxa, the relative size of the olfactory bulbs (OBs) is highly variable, ranging from about 1.9% respect to the whole brain size in North Island brown kiwi (*Apteryx mantelli*) [17] to 0.03% in carrion crow (*Corvus corone*) [18]. Passeriformes are among the avian orders with relatively smaller OBs [19,20]. In particular, house sparrows (*Passer domesticus*) have a relative size of the olfactory bulbs respect to the telencephalon (OBs excluded) of about 0.08% [18]. Hence, it is not surprising if they have never been investigated for their olfactory sensitivity. However, predator avoidance mediated by chemical cues varies greatly among species. For example, blue tits (*Cyanistes caeruleus*) have been shown to recognize the chemical cues of a predator like a male ferret (*Mustela furo*) and subsequently demonstrated antipredatory behaviours to decrease predation risk when foraging [12]. Avoidance of predator odour cues was also shown in great tits (*Parus major*) [13]. Similar results were obtained in domestic fowls (*Gallus gallus domesticus*) and house finches (*Haemorhous mexicanus*) [14,15], supporting the hypothesis that birds can detect predator proximity through odorous cues. On the contrary, predator avoidance mediated by chemical cues is not supported in Eastern bluebirds (*Sialia sialis*) and house wrens (*Troglodytes aedon*), where there was no difference in the exploratory behaviour of nest boxes with or without predator scent [21,22].

Detecting predator signs or activity may be particularly advantageous when it comes to evaluating and choosing future breeding sites. However, olfactory assessment of nest site features may not be restricted to evaluating the risk of offspring or adult predation. Given that some avian species incorporate aromatic plants as nest material [23–25], other odour related nest quality features might be important as well. It has been shown that starlings (*Sturnus vulgaris*) and blue tits use biologically active aromatic plants [23,25]. This odorous nesting material may be able to reduce ectoparasites and pathogen populations as suggested by the nest protection hypothesis [26,27].

Evidence for odour related nest site assessment is often found in burrowing and hole-nesting species where visual cues cannot be used to evaluate possible threats [28,29]. However, olfaction is used in similar conditions also in non-exclusively hole nesting specie as zebra finches (*Taeniopygia guttata*) and Bengalese finches (*Lonchura striata var*. *domestica*) [30]. Olfactory evaluation of the nest site might also be related to competition, which can even result in brood demolition or predation [31–33]. In a natural environment, one of the most dangerous times of the day is probably the night when rodents are mainly active [34,35]. Furthermore, another recent study suggests that birds are not able to detect predator chemical cues while sleeping [36]. Hence, olfactory detection of competitor and predator cues in the nesting or roosting area might be especially advantageous.

Therefore, this study aimed to investigate whether a possible competitor or predator is recognized by a hole-nesting passerine, namely the house sparrow. We tested the olfactory perception of two distinct and possibly nest site relevant odours. House sparrows are anthropophilous, commonly found in cities, farms and zoos with high abundance of artificial feeding sources [37,38]. This also applies to mice, in particular the common house mouse (*Mus musculus domesticus*), which uses similar environments and represents a potential competitor for sparrows, occupying bird nest boxes (authors’ pers. observ.). In addition to that, mice prey on eggs and hatchlings and might pose a significant risk to breeding house sparrows [31–33,37]. In particular, choosing a nest site periodically frequented by mice is likely to cause the loss of the whole brood. Moreover, adult house sparrows may be injured by mice competing for the same place. However, it should be noted that house sparrow’s eggs and nestlings can also be predated by different species such as beech martens (*Martes foina*), pine martens (*Martes martes*) or Aesculapian snakes (*Zamenis longissimus*). To test whether sparrows avoid the presence of mice, male and female sparrows were exposed to odours of hay and mouse urine. Hay has a distinctive smell. Hay is commonly used by house sparrows to build nests and represents a common”not negative” or even positive odour. In fact, hay is the prevalent nest material and is sometimes used in male displaying behaviour [38]. Thus, we considered this scent to be familiar and innocuous. We hypothesized that house sparrows may perceive and respond to different environmental cues. The experiment was performed at the sunset to simulate a first inspection to new possible roosting or nesting sites. In particular, individuals may spend more time near the hay odour choosing a safe roosting or nesting environment. Depending on the species there might be also sex specific differences in nest site selection. In the house sparrow, as for many passerine species, the first exploration of possible nesting sites is made by males (authors’ pers. observ.), and then the selected area is offered to the female. Hence, males likely face a higher risk when inspecting new unexplored sites. Thus, one may even predict sex specific differences in the ability or sensibility towards nest site related odours and males may be more sensitive to odour-related nest site features. Our experiment is therefore also designed to simulate a first inspection to new possible roosting or nesting sites.

## Materials and Methods

### Ethical note

Housing conditions as well as the capture and handling of house sparrows conformed with the Austrian laws and were licensed by the government of Vienna (MA22-231/2011). The Ethical Commission of the University of Veterinary Medicine of Vienna (Austria) approved the experiments in accordance with Good Scientific Practice guidelines and national legislation (68.205/107-WF/V/3b/2016).

The condition and health of experimental birds were monitored on a daily basis.

### Housing and study species

The study was conducted between March and April at the Konrad Lorenz Institute of Ethology (KLIVV, University of Veterinary Medicine) in Vienna, Austria (48°13′ N, 16°17′ E). The house sparrows *Passer domesticus* (Linnaeus, 1758) used in the experiments are the third generation of a captive population [39]. All birds were born in the previous year and had no breeding experience. Experimental trials ended just before the onset of house sparrows’ breeding season (authors’ pers. observ.). Housing conditions allowed the sparrows to have manifold contacts with hay and house mice urine during the year. Hence, we assumed that all tested individuals were familiar to the olfactory stimuli offered in the experiment. The birds were kept in mixed-sex outdoor aviaries (measuring 2 × 3.9 m and 2.6 m high) provided with wooden nest boxes and received food (a mixture of millet, canary seeds, wheat, sunflower seeds, protein-based mash and apple slices) and water ad libitum [40]. No bird tried to nest in the outdoor aviaries before or during the experiment.

### Experimental procedure

Individuals were tested in an experimental cage (cage model: Montana Terenzo; 1 × 0.5 × 0.5 m) in a separated room (6.60 × 3.30 × 2.25 m). To provide standardised conditions and correct for side preferences, the cage was equipped with four identical feeders (9 × 8 × 5 cm), four wooden perches (50 cm long) and two plastic nest boxes (23 × 15 × 12 cm; entrance hole diameter: 5 cm). We used three rooms, three cages, and twelve nest boxes in total. Every day, half of the nest boxes were used in the experimental set-up while the remaining half was carefully cleaned. Every cage was singularly placed in a room.

All nest boxes were equipped with a stick (17 cm long) underneath the entrance hole. All sticks were obtained from leafless branches of the same local tree. The three experimental rooms were maintained at a temperature of about 18–20°C. They were visually isolated from the outside. Only artificial light was provided with light cycles mimicking the natural light/dark cycle for the date. Sunset was reproduced through sequential switching of four white neon lights (single switcher), a 40W white lamp and a blue neon light every 30 min. Trials started 75 min before turning the last lamp off (i.e. 15 min before the start of artificial sunset). Hence, birds were free to explore the experimental set-up in full light for at least 15 min before the start of artificial sunset. As neon lights reproduced the natural light/dark cycle, the starting time of each trial followed the natural sunset time during the season. During the experiment, commercial food for granivorous passerines and water was provided ad libitum.

We offered two different odours in the nest boxes, to males and females, namely the scent of mouse urine and the odour of hay. The two nest boxes were visually identical, internally and externally (see below), so the only difference was the odour contained. In order to collect mouse *Mus musculus domesticus* (Linnaeus, 1758) urine scent, we placed 13 cm^2^ (3.5 × 3.7 cm) pieces of drawing paper in a plastic bag with wood shavings soaked with mouse urine (mice bedding). The bedding came from ten males housed singularly at the KLIVV. No mice had ever been in a social setting and hence they have never been able to establish a dominance hierarchy. Wood shavings were stored at -20°C to reduce bacterial growth possibly affecting the odorous characteristics. After 24 h, we collected and used the sheets to scent experimental nest boxes by attaching them at the box entrance holes. Moreover, to strengthen the competitor odorous stimulus, we placed 17 g of wood shavings soaked with mouse urine inside one nest box. Shavings were covered with 2 g of cotton wool in order to avoid visual interference. In the other nest box (hay odour stimulus), we put 5 g of hay and covered it with 2 g of odourless cotton wool. A 13 cm^2^ sheet was stored for 24 h in the hay before being attached to the respective nest box entrance. Rooms were ventilated every day for at least 60 min after the removal of chemical cues. Cages and nest boxes were well washed with hot water (about 70°C), dried with unscented kitchen roll, and cleaned with 70% Ethanol. The bottoms of the cages were covered with brown wrapping paper to ease cleaning. We removed and cleaned the nest boxes, sticks, and perches with hot water on a daily basis. The cages were washed and the wrapping paper was removed every two days. We used latex examination gloves during cleaning protocols and preparation of all odorous stimuli. The position of the different odorous stimuli, nest box identity and experimental room number were randomised at every trial using a random number generator [41]. The nest boxes in every treatment had a couple of hay stems partially protruding from the entrance hole to stimulate exploration and improve the naturalness of the experimental set-up.

We tested 15 females and 15 males, each focal bird was caught in late morning (11.30–12.30 a.m.). Immediately after capture, the sparrow was introduced into an acclimatisation cage and given about four hours to acclimatise. Then, sparrows were individually moved to the experimental cages. We video recorded the behaviour of sparrows in the experimental cage using iSpy, v. 6.3.0.0 an open source software (www.ispyconnect.com). Videos were analysed by one experimenter who was blind to the subject identity and position of the odorous cues while scoring. Similarly to Amo et al. [13] and Krause and Caspers [30], we defined a visit to an odorous stimulus when a bird perched on the stick located beneath the entry hole of a nest box or entered inside the nest box itself (choice area). As choice time, we used the total time spent by each sparrow in the choice area (i.e. on the stick in front of the nest box entrance or inside the nest boxes). An analogous data analysis was followed by Krause and Caspers [30]. When house sparrows were inside this area, we considered the individuals as motivated to explore the nest boxes and possibly smell their chemical cues. Choice time was measured in seconds, using the timer superimposed upon video recordings by iSpy software. Preference was expressed as the proportion of time in front of each odorous stimulus over the total time in the choice area.

### Repeatability of individual preference for odour

A basic assumption for our experiment on odour preference is some level of consistency in individual preference [42,43]. For this reason, for a subsample of individuals, we repeated the experiment with the same individuals the day after each test to investigate the consistency of individual house sparrows’ choices. Briefly, the same individuals (males: n = 9, females: n = 11), were tested two times, using the same experimental set-up described above. The position of the two stimuli was switched after each experiment to exclude any effect of preference for positions. We determined the repeatability of each individual’s preference for a particular odour (mouse urine or hay) between two trials following Lessells and Boag [44], by calculating separate analyses of variance for each focal individual with association time as the dependent variable and trial number as an independent variable. We found a high consistency of male preference, but female consistency was low (repeatability of time allocation by a male tested twice: R = 0.61, SE = 0.22; repeatability of time allocation by a female tested twice: R = 0.32, SE = 0.28).

### Statistical analysis

Statistical analyses were performed with IBM SPSS Statistics, v. 22.0 (IBM, Armonk, NY, U.S.A.). All tests are two-tailed and all the results are presented as mean ± SE. Proportions were arcsine square root transformed before the analyses [45]. Analyses were checked to ensure that they met the assumptions of parametric statistics. Individual preference for a particular odour was analysed with a paired t-test. To test for differences in time spent in the choice area between males and females, percentages of time were standardised for normalisation and then analysed with a two-sample t-test and an ANOVA test.

## Results

**Fig 1:**
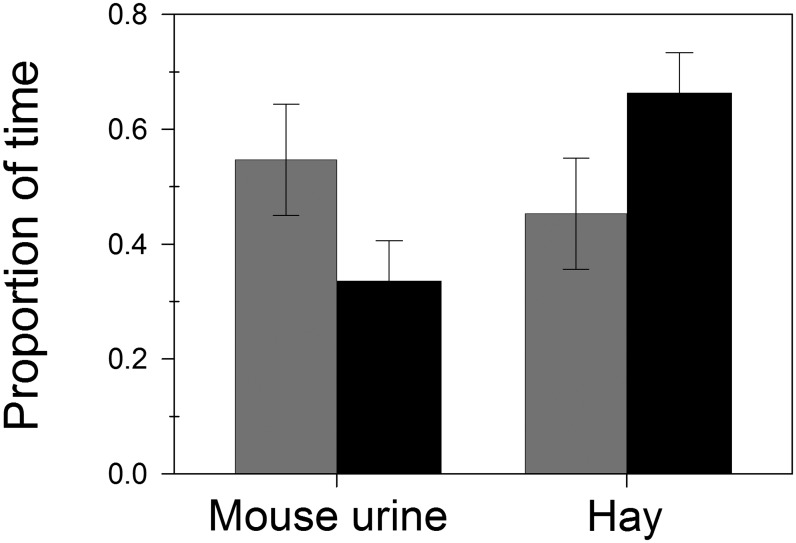
Males’ and females’ proportion of time in front of the odorous stimuli. Relative time spent by males (black bars, n = 15) and females (grey bars, n = 14) in front of a nest box containing mouse urine scent or hay odour. Only time in the choice area was considered. Males spent significantly more time in front of hay odour (paired t-test: t_14_ = 2.182, P = 0.047) while females do not show any significant preference between the two different stimuli (paired t-test: t_13_ = 0.482, P = 0.638). Figure shows non-transformed data. Error bars indicate ± SE.

All males visited both nest boxes, while one female was excluded from the analyses due to impaired exploratory behaviour. Males and females spent on average respectively 15% and 8% of the total experimental time in the choice area. However, this difference was not statistically significant (Student t-test: t_27_ = 1.542, P = 0.135). Comparing the time spent at the two different odours, males spent significantly more time in front of the hay odour respect to the mouse urine scent (paired t-test: t_14_ = 2.182, P = 0.047, Fig 1 black bars). In contrast, females did not show any clear preference for the hay smell (paired t-test: t_13_ = 0.482, P = 0.638, Fig 1 grey bars). The treatment (stimulus) × sex interaction was marginally not significant (ANOVA: F_1,27_ = 2.955, P = 0.097). Finally, no significant differences in the exploratory behaviour (i.e. exploration time and nest site preference) were found between the full light and artificial sunset period during the trials (males: P > 0.57; females: P > 0.49).

## Discussion

This is the first evidence for the use of odour cues in house sparrows and the results obtained in this context suggest that the use of odour cues in birds may be sex specific. Male house sparrows spent significantly more time in front of nest boxes provided with the hay odorous stimulus than the mouse urine, whereas females did not display a clear preference. This may indicate that during the initial inspection of a new possible nest site, males but not females avoid the scent of mice. One possible explanation is that in house sparrows, the males are the ones who choose the nest site [38]. In this context, males have to inspect new unexplored sites where they may be more likely facing a higher risk of predation, injuries or encountering competitors. Hence, males may have developed a more refined ability or susceptibility than females towards odours related to nesting sites. Our experimental study is in accordance and strengthen Krause and Caspers [30] conclusions indicating that the use of olfactory cues may be sex-specific. Own observations suggest that house mice are usually more successful and dominant competitors in occupying nest boxes and force house sparrows to desert the nest box even in advanced breeding stages (own observation). Furthermore, the presence of mice might pose an increased risk of predation or pathogen exposure to eggs and hatchlings. In contrast, hay and its odour, which represents the major component of house sparrow nests, might play a role in male displaying behaviour [38] rather than representing a neutral odour only. Thus, theoretically, several odour-related nest site features might be integrated in nest site choice. To our knowledge, no data is available on bird preference for clean nest material (in this case hay). Conversely, many studies show avian avoidance for the scent of predators [12–15]. Moreover, nest material is usually easily available in nature and the evolution of a specific preference for hay odour should be considered unlikely. Furthermore, it can be hypothesized that hay is rarely a good indicator of site occupancy by a conspecific since vocal calls and specific body postures are commonly used to proclaim ownership at nest site [37]. These behaviours are generally far more clear to signal site occupancy than hay alone. Moreover, house sparrows usually remain in the same colony for a great part of their life breeding in the same nest site for many years [37]. Differently, competitor avoidance through olfactory cues can improve hatching success and give a substantial advantage to its bearer. Hence, even if it is not possible to rule out the possibility that males were driven in their choice by preference for the hay odour, we think that the avoidance of mouse urine rather than the preference for hay may be the major driving force responsible for the outcome of the results of this study. No significant differences in the exploratory behaviour (i.e. exploration time and nest site preference) were found between the full light and artificial sunset period during the trials.

Our findings are in line with similar results on exclusively hole-nesting species, like blue tits and great tits, which displayed behaviours associated with predator avoidance when exposed to faeces of ferrets [12,13]. Furthermore, red junglefowls (*Gallus gallus*) were more vigilant and searched less for food when exposed to the scent of tiger (*Panthera tigris*) and dhole (*Cuon alpinus*) faeces [15]. In addition, house finches generally avoid feeders with mammalian smell [14]. We therefore suggest that the preference exhibited by male house sparrows can be considered adaptive. Moreover, when it comes to evaluating for a possible nest site and its hazards multiple sensory modes are likely to be involved. Birds are regarded to mainly rely on visual cues but single sensory modes are error prone. Conversely, the use of a multi-modal way of assessment might reduce erroneous evaluations and be favoured by selection [30]. In fact, choosing a safe nesting or roosting site could be crucial to avoid predation [36]. Furthermore, the scent of faeces and urine might be associated with a potential source of bacteria and parasites, indicating an environment containing pathogens possibly affecting adults, eggs, or the offspring [14].

In contrast to many other experiments, we did not use a Y-maze or T-maze apparatus [9,13,46,47]. In our experimental set-up, house sparrows were free to explore the experimental environment and to choose between different chemical stimuli. Moreover, individuals were not surgically treated, thus avoiding high stress levels and collateral modifications to other behaviours unrelated to odour.

In conclusion, our results strengthen the hypothesis that oscines use olfaction as reliable information to assess the environment. In particular, male house sparrow avoidance of mouse urine scent just shortly before the onset of the breeding season could be adaptive when choosing a safe environment for breeding and roosting.

## References

[1] Dell'Ariccia G, Bonadonna F. Back home at night or out until morning? Nycthemeral variations in homing of anosmic Cory's shearwaters in a diurnal colony. J Exp Biol. 2013; 216: 1430–1433. 10.1242/jeb.082826 23307801

[2] Gagliardo A. Forty years of olfactory navigation in birds. J Exp Biol. 2013; 216: 2165–2171. 10.1242/jeb.070250 23720797

[3] Grubb TC. Olfactory navigation to the nesting burrow in Leach's petrel (*Oceanodroma leucorrhoa*). Anim Behav. 1974; 22: 192–202. 4836743 10.1016/s0003-3472(74)80069-2

[4] Amo L, Jansen JJ, Dam NM, Dicke M, Visser ME. Birds exploit herbivore-induced plant volatiles to locate herbivorous prey. Ecol Lett. 2013; 16: 1348–1355. 10.1111/ele.12177 24103093

[5] Hutchison LV, Wenzel BM. Olfactory guidance in foraging by procellariiforms. Condor. 1980; 82: 314–319.

[6] Nevitt GA. Olfactory foraging by Antarctic procellariiform seabirds: life at high Reynolds numbers. Biol Bull. 2000; 198: 245–253. 10.2307/1542527 10786944

[7] Nevitt GA. Sensory ecology on the high seas: the odor world of the procellariiforms seabirds. J Exp Biol. 2008; 211: 1706–1713. 10.1242/jeb.015412 18490385

[8] Amo L, López-Rull I, Pagán I, Macías Garcia C. Male quality and conspecific scent preferences in the house finch, *Carpodacus mexicanus*. Anim Behav. 2012; 84: 1483–1489.

[9] Bonadonna F, Nevitt GA. Partner-specific odor recognition in an Antarctic seabird. Science. 2004; 306: 835 10.1126/science.1103001 15514149

[10] Bonadonna F, Sanz-Aguilar A. Kin recognition and inbreeding avoidance in wild birds: the first evidence for individual kin-related odour recognition. Anim Behav. 2012; 84: 509–513.

[11] Krause ET, Kruger O, Kohlmeier P, Caspers BA. Olfactory kin recognition in a songbird. Biol Lett. 2012; 8: 327–329. 10.1098/rsbl.2011.1093 22219391 PMC3367747

[12] Amo L, Galván I, Tomás G, Sanz JJ. Predator odour recognition and avoidance in a songbird. Funct Ecol. 2008; 22: 289–293.

[13] Amo L, Visser ME, Oers Kv. Smelling out predators is innate in birds. Ardea. 2011; 99: 177–184.

[14] Roth TC, Cox JG, Lima SL. Can foraging birds assess predation risk by scent? Anim Behav. 2008; 76: 2021–2027.

[15] Zidar J, Løvlie H. Scent of the enemy: behavioural responses to predator faecal odour in the fowl. Anim Behav. 2012; 84: 547–554.

[16] Bang BG. Anatomical evidence for olfactory function in some species of birds. Nature. 1960; 188: 547–549. 13686568 10.1038/188547a0

[17] Corfield JR, Wild JM, Parsons S, Kubke MF. Morphometric analysis of telencephalic structure in a variety of neognath and paleognath bird species reveals regional differences associated with specific behavioural traits. Brain Behav Evolut. 2012; 80: 181–195. 10.1159/000339828 22890218

[18] Mehlhorn J, Hunt GR, Gray RD, Rehkamper G, Gunturkun O. Tool-making New Caledonian crows have large associative brain areas. Brain Behav Evolut. 2010; 75: 63–70. 10.1159/000295151 20215728

[19] Bang BG, Cobb S. The size of the olfactory bulb in 108 species of birds. Auk. 1968; 85: 55–61.

[20] Corfield JR, Price K, Iwaniuk AN, Gutierrez-Ibañez C, Birkhead T, Wylie DR. Diversity in olfactory bulb size in birds reflects allometry, ecology, and phylogeny. Front Neuroanat. 2015; 9: 102 10.3389/fnana.2015.00102 26283931 PMC4518324

[21] Godard RD, Bowers BB, Morgan Wilson C. Eastern bluebirds *Sialia sialis* do not avoid nest boxes with chemical cues from two common nest predators. J Avian Biol. 2007; 38: 128–131.

[22] Johnson LS, Murphy SM, Parrish GW. Lack of predator-odor detection and avoidance by a songbird, the House Wren. J Field Ornithol. 2011; 82: 150–157.

[23] Clark L, Mason JR. Effect of biologically active plants used as nest material and the derived benefit to starling nestlings. Oecologia. 1988; 77: 174–180. 28310369 10.1007/BF00379183

[24] Gwinner H, Berger S. Starling males select green nest material by olfaction using experience-independent and experience-dependent cues. Anim Behav. 2008; 75: 971–976.

[25] Petit C, Hossaert-McKey M, Perret P, Blondel J, Lambrechts MM. Blue tits use selected plants and olfaction to maintain an aromatic environment for nestlings. Ecol Lett. 2002; 5: 585–589.

[26] Clark L. The nest protection hypothesis: the adaptive use of plant secondary compounds by European starlings In: Loye JE, Zuk M, editors. Bird-parasite interactions: ecology, evolution and behaviour. Oxford: Oxford University Press; 1991 pp. 204–221.

[27] Wimberger PH. The use of green plant material in bird nests to avoid ectoparasites. Auk. 1984; 101: 615–618.

[28] Bonadonna F, Cunningham GB, Jouventin P, Hesters F, Nevitt GA. Evidence for nest-odour recognition in two species of diving petrel. J Exp Biol. 2003; 206: 3719–3722. 12966063 10.1242/jeb.00610

[29] Bonadonna F, Bretagnolle V. Smelling home: a good solution for burrow-finding in nocturnal petrels? J Exp Biol. 2002; 205: 2519–2523. 12124375 10.1242/jeb.205.16.2519

[30] Krause ET, Caspers BA. Are olfactory cues involved in nest recognition in two social species of Estrildid finches? PLoS ONE. 2012; 7: e36615 10.1371/journal.pone.0036615 22574196 PMC3344906

[31] Blight LK, Ryder JL, Bertram DF. Predation on rhinoceros auklet eggs by a native population of *Peromyscus*. Condor. 1999; 101: 871–876.

[32] Bradley JE, Marzluff JM. Rodents as nest predators: influences on predatory behavior and consequences to nesting birds. Auk. 2003; 120: 1180–1187.

[33] DeGraaf RM, Maier TJ, Fuller TK. Predation of small eggs in artificial nests: effects of nest position, edge, and potential predator abundance in extensive forest. Wilson Bull. 1999; 111: 236–242.

[34] Picman J, Schriml LM. A camera study of temporal patterns of nest predation in different habitats. Wilson Bull. 1994; 106: 456–465.

[35] Thompson FR III, Dijak W, Burhans DE. Video identification of predators at songbird nests in old fields. Auk. 1999; 116: 259–264.

[36] Amo L, Caro SP, Visser ME. Sleeping birds do not respond to predator odour. PLoS ONE. 2011; 6: ID e27576 10.1371/journal.pone.0027576 22110676 PMC3217974

[37] Cramp S, Perrins CM. Handbook of the birds of Europe, the Middle East and North Africa The birds of the Western Palearctic, vol. VIII: Crows to finches. Oxford: Oxford University Press; 1992.

[38] Summers-Smith JD. The house sparrow. London: Collins, New Naturalist; 1963.

[39] Griggio M, Biard C, Penn DJ, Hoi H. Female house sparrows “count on” male genes: experimental evidence for MHC-dependent mate preference in birds. BMC Evol Biol. 2011; 11: 44 10.1186/1471-2148-11-44 21320306 PMC3044665

[40] Griggio M, Hoi H. Only females in poor condition display a clear preference and prefer males with an average badge. BMC Evol Biol. 2010; 10: 261 10.1186/1471-2148-10-261 20799928 PMC2939576

[41] Haahr M. True random number service; 1998.

[42] Forstmeier W, Birkhead TR. Repeatability of mate choice in the zebra finch: consistency within and between females. Anim Behav. 2004; 68: 1017–1028.

[43] Griggio M, Hoi H, Lukasch B, Pilastro A. Context-dependent female preference for multiple ornaments in the bearded reedling. Ecol Evol. 2016; 6: 493–501. 10.1002/ece3.1903 26843933 PMC4729251

[44] Lessells CM, Boag PT. Unrepeatable repeatabilities: a common mistake. Auk 1987; 104: 116–121.

[45] Sokal R, Rohlf F. Biometry. 3rd ed New York: WH Freeman and Company; 1995.

[46] Bonadonna F, Hesters F, Jouventin P. Scent of a nest: discrimination of own-nest odours in Antarctic prions, *Pachyptila desolata*. Behav Ecol Sociobiol. 2003; 54: 174–178.

[47] Hagelin JC, Jones IL, Rasmussen LE. A tangerine-scented social odour in a monogamous seabird. Proc R Soc Lond B Biol Sci. 2003; 270: 1323–1329. 10.1098/rspb.2003.2379 PMC1691389 12965022

